# Keeping Bystanders Active: Resuscitating Resuscitation Skills

**DOI:** 10.3389/fpubh.2019.00177

**Published:** 2019-06-27

**Authors:** Sarah C. Maaß, Florian Sense, Kevin A. Gluck, Hedderik van Rijn

**Affiliations:** ^1^Department of Experimental Psychology, University of Groningen, Groningen, Netherlands; ^2^Behavioral and Cognitive Neurosciences, University of Groningen, Groningen, Netherlands; ^3^Air Force Research Laboratory, Wright-Patterson Air Force Base, Dayton, OH, United States

**Keywords:** cardiopulmonary resuscitation, active bystanders, long-term retention, low-intensity retraining, general population proficiency, video and simulation training

## Abstract

**Introduction:** Sufficient CPR skills in the general population are essential to make them active bystanders and contribute to an effective chain of survival in cardiac arrest emergencies. However, having a large proportion of the population regularly retrained is practically infeasible.

**Objective:** The aim of this study was to assess and retrain cardiopulmonary resuscitation (CPR) skills of individuals who received (limited) CPR training several months to years prior.

**Method:** Ninety-nine German adults in a possession of a driver's license were asked to perform CPR on a Laerdal Resusci Anne^®^ QCPR manikin (Laerdal, Stavanger, Norway). After initial assessment, participants watched an instructional video and completed short, isolated compression, and ventilation practice with live feedback. CPR competency was assessed again after retraining and after a retention interval of 45 min.

**Results:** Our results indicate that only 2% of participants managed to reach the performance criteria set by the European Resuscitation Council Guidelines, with most failing to reach even the lowest levels of performance. This corroborates earlier observations that CPR skills have deteriorated almost completely after a long retention interval, calling into question “one-and-done” certification of this basic life-saving. However, we also demonstrated that performance strikingly increased after watching a 6-min instructional video and a short opportunity for isolated practice. This increase in performance was stable over 45 min with 96% of participants meeting performance levels specified in the Guidelines. Closer inspection of the isolated compression practice data suggests that performance was very high at the start of the practice already, indicating that short refresher videos might suffice to change bystanders that would not have initiated CPR due to lack of knowledge into active first responders.

**Conclusion:** We suggest that short refresher trainings could be an effective and affordable means of improving basic lifesaving skills to increase the effective contribution of bystanders during emergencies.

## Introduction

With 70,000 to 80,000 individuals per year experiencing sudden cardiac arrest in Germany, even without major, wide-scale emergencies, this condition is among the country's most frequent causes of death. As the time between cardiac arrest and starting cardiopulmonary resuscitation (CPR) is critical for survival, effective bystander CPR is a key determinant of the survival rate of cardiac arrest victims (1). Sufficient CPR skills in the general population are essential for an effective chain of survival (2). Even though CPR-trained bystanders might still fail to perform CPR (3), lacking even basic CPR knowledge makes it impossible for any bystander to actively contribute in cases of emergencies: Even though bystanders might not meet the proficiency levels of skilled medical personnel, attempting to start CPR is better than inactivity among bystanders (4).

In Germany, a large percentage of the adult population has been trained in resuscitation [over 80% in a sample of 200 participants (5)] because completing CPR training is part of the requirements for obtaining a driver's license. As this is a one-session First Aid certification, including CPR training, no retraining, or recertification is formally required. However, given the level of competence that can be expected at population level, it is surprising that an alarmingly low percentage of people provide First Aid care, and more specifically, initiate CPR before Emergency Medical Services arrive (5). Given the critical role of bystanders in minimizing the consequences of a cardiac arrest, a role that would be even more critical in the case of large-scale emergencies, this discrepancy between a potentially well-trained population and low levels of CPR assistance is worth investigating.

As CPR skills are known to deteriorate over time (6), it could be that the CPR training that is part of the one session First Aid certification is not sufficient for long-term competency. This idea is supported by the observation that even in medical professionals, performance typically deteriorates in the first year after certification (7, 8). Furthermore, in a recent German study, only 40% of a general-public sample reported they were capable of initiating CPR, as measured via a self-assessment questionnaire (5). This percentage increased markedly after a short refresher training was provided. No quantitative performance data were reported in that study, but it suggests that additional instruction at least increases subjectively perceived CPR skills.

### Objectives

To quantify the competence of CPR skills in the general population and the potential effectiveness of a short, low-intensity refresher training to reduce any knowledge-based constraints in performing CPR, we first assessed the level of CPR performance that was retained over months to years after a first-aid course. Second, we assessed the increase in performance after a short training video used to restore CPR competency to European Resuscitation Council Guideline standards (9).

## Methods

For this study, we invited 99 German participants (age 18–27, *M* = 20.8, enrolled as students at the University of Groningen, The Netherlands) with valid German driver's licenses. The time since obtaining their driver's license ranged from less than a month to more than 10 years prior to participation (*M* = 45.8, *SD* = 26.05, in months). All participants gave written informed consent and ethical approval was obtained to conduct the study (WIRB protocol #20151567).

Immediately after arriving for the study, participants underwent an initial skill assessment in which they performed CPR on a Laerdal Resusci Anne^®^ QCPR manikin (Laerdal, Stavanger, Norway). Performance was recorded using a SimPad PLUS device (Laerdal, Stavanger, Norway) with activated SkillReporter software, without any form of feedback. After this initial assessment, all participants watched a 5:46 min instructional video (see https://osf.io/w5ze2) in which the basic CPR procedure, following European Resuscitation Council Guidelines 2015 (8), was explained and demonstrated. Immediately after this video, they were asked to perform compressions and rescue breaths independently for 1 min each, with the manikin's live-feedback option enabled on the SimPad and thus providing immediate feedback to the participant. After these training sessions, CPR performance was re-assessed in two tests. During these tests, no feedback was provided, but a certified CPR trainer provided personalized feedback and instruction in between these tests when necessary. After a delay of 45 min, in which participants performed a number of unrelated tasks, CPR skills were assessed again using the SimPad device, without feedback. Note that this study was embedded in a multi-session experiment, details of which will be reported in a separate manuscript (see https://osf.io/zpjrt/ for project details).

## Results

**Figure 1:**
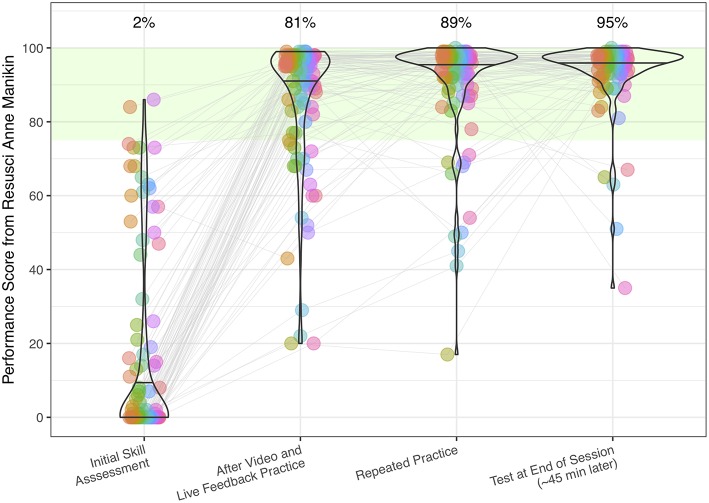
Individual participants' (*n* = 99) composite CPR performance scores and violin plots of the sample distribution, measured with the Laerdal Resusci Anne QCPR manikin (Laerdal, Stavanger, Norway) during four test sessions. Green area indicates performance above the minimum competency level (8). Numbers above the violin plots indicate percentage of participants performing this skill above the minimum required competency level of 75.

On the initial skill assessment, 78.4% of participants did not attempt to administer rescue breaths and 6.2% did not perform a single compression (i.e., did not initiate CPR at all). Even though most participants did initiate chest compressions, the vast majority ceased chest compressions too early: The average number of compressions was 48.2, with 23.7% administering less than 20 and 83.5% less than 90 compressions. This resulted in the extremely poor overall CPR performance on the initial skill assessment evident for many participants in Figure 1: 50.5% of participants scored 0 and 75.3% scored below 20. That is, participants' initial performance was far below minimum competency levels, with only 2% of participants meeting the criteria set by the European Resuscitation Council Guidelines of 2015 (8) (first column in Figure 1).

Interestingly, skill performance improved strikingly after watching the instructional video and performing compressions and rescue breaths for 1 min, with 81% of participants passing the criteria set by the European Resuscitation Council after this short refresher (second column in Figure 1). Personalized feedback further improved performance (third column, Figure 1), a performance level which seemed to be stable when retested after 45 min (fourth column, Figure 1). Importantly, participants also markedly improved with respect to the required steps that have to be performed before executing CPR, which are listed in the four top-most rows of Table 1.

**Table 1:** CPR skill assessment scores^a^.

	**Initial skill assessment (%)**	**Assessment after video and 1 min training sessions (%)**	**Second assessment after video and personalized instruction (%)**	**Test after 45 min (%)**
Check response	55	100	100	99
Call for help	62	93	97	96
Open Airway	5	82	94	95
Breathing check	71	92	97	98
Proper hand position	91	99	100	99
Compression	11	85	91	96
Ventilation	7	89	92	94

a *Percentages indicate proportion of subjects performing satisfactorily (criteria described above). For Check responsiveness, Call for help, Open airway, and Breathing check the scoring was binary (correct/incorrect scored by a certified CPR instructor) and the data in the table show the % of participants who did the action correctly. For compression and ventilation, the percentages indicate the proportion of subjects reaching the criterion of 75% in skill performance recorded with the SimPad PLUS device (Laerdal, Stavanger, Norway). Note that Proper hand position, Compression and Ventilation are part of the composite score reported in Figure 1*.

**Figure 2:**
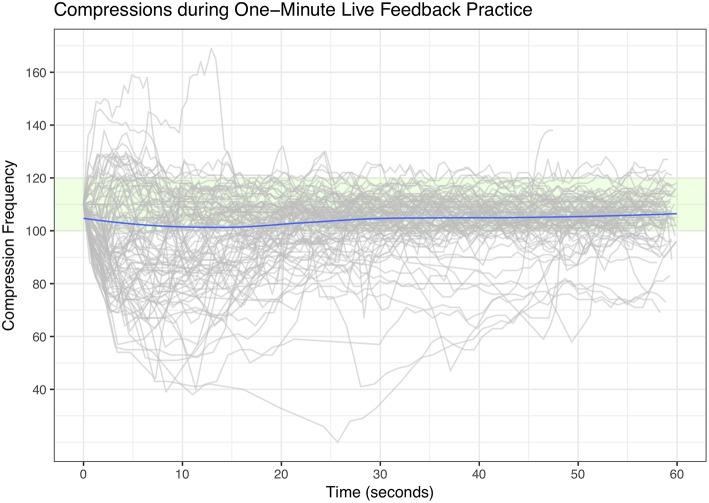
Compression frequency (in compressions/minute) per participant as recorded during the 1-min training session immediately after watching the instructional video. Green area indicates performance in line with set criteria (9). Blue line indicates average performance at each point in time.

As suggested previously by Van Berkom et al. (10), there may be some cause for concern regarding the composite performance score generated by the embedded measurement system. However, the measurement system on the manikin also records the separate measures comprising the composite score—such as compression rate, compression depth, release height, and hand position—, which can be analyzed instead of the proprietary composite score. Analyses of these separate measures indicate that performance at the start of the training sessions was already at the performance level required by the European Resuscitation Council Guidelines, indicating that watching the short video, without hands-on training, improved CPR performance. This can be seen in Figure 2 in which performance over time during the compression practice training is plotted for each participant separately (each line in the plot represents the values recorded for a single participant). This figure demonstrates that only a handful of participants completely fail to reach the required compression frequency, and that most achieve the 104 compressions per minute demonstrated in the video. Moreover, even though a proportion of the participants require the live-feedback to increase their compression rate to ERC criteria (9), most participants immediately start out with reasonable compression rates. We refer the interested reader to the supplementary analyses (https://osf.io/zpjrt/), which contains equivalent visualizations of other subscores recorded by the manikin. In summary, watching a short instructional video sufficed to retrain either inactive or ineffective bystanders.

## Discussion

The results of this study demonstrate that: (1) After a long retention interval, even young, highly educated individuals are not “ready, willing and able to act” (11) when confronted with a simulated victim of a cardiac arrest, but that (2) Watching a short instructional video, potentially combined with short practice sessions including feedback, restores performance for the majority of participants. Combined, this work suggests that providing one-shot training to young adults is not sufficient for them to retain basic lifesaving skills which could be required in cases of an emergency. However, merely presenting an instructional video that refreshes previously trained skills may be sufficient to regain competence in these basic lifesaving skills for the majority of participants, even years after initial training. These findings are especially relevant if there already is an infrastructure to provide initial training to the general population (like in Germany), and were dormant skills could be efficiently retrained to reach widespread CPR competence.

These results also highlight the limited effectiveness of a single training session, when not followed up by later refresher trainings. “One-and-done” certification practices are antiquated and unresponsive to contemporary basic and applied cognitive science (12). The data presented here also suggest that the proportion of potential bystanders that actually initiate and perform effective CPR (as low as 2%, see Figure 1) might be much lower than the number of people that self-report that they would initiate CPR [40% according to (5)]. Our results are consistent with some previously published research (13) suggesting that refresher training can be of much lower intensity, as people with previous training relearn more effectively (14). The effectiveness of watching an instructional video, as reported here, strongly argues for making brief refresher training easily accessible. That video training can be as effective as instructor-led training has been demonstrated in earlier work (15), in which a 30-min video-based self-training provided positive results. Interestingly, our work demonstrates that a much shorter, 6-min video clip can also result in proficient CPR performance. Future research should establish how long the benefits from a short refresher video persist and whether they are equally pronounced in a broader sample.

Providing access to short but effective (re)training will probably lower the threshold for self-initiation of training or would make it more likely that people would follow-up on externally provided reminders for self-training (16). Given the almost perfect coverage of CPR training in German adults, one could argue that such instructional videos should be made available via screens in public spaces or be part of national broadcasting services. If these videos were attended regularly, the resuscitation skills of an entire population could be continuously resuscitated, increasing the chances that bystanders will act effectively in case of a cardiac arrest in emergencies, potentially saving thousands of lives in the process.

## Ethics Statement

This study was carried out in accordance with the recommendations of the Western Institutional Review Board (WIRB) with written informed consent from all subjects. All subjects gave written informed consent in accordance with the Declaration of Helsinki. The protocol was approved by the WIRB.

## Author Contributions

SM, FS, KG, and HR contributed to the conception and design of the study, the planning of the analyses, and the interpretation of the results. SM performed the data collection. FS organized the database and performed the statistical analyses. SM and FS wrote the first draft of the manuscript. KG and HR wrote sections of the manuscript. All authors contributed to manuscript revision, read, and approved the submitted version.

### Conflict of Interest Statement

The authors declare that the research was conducted in the absence of any commercial or financial relationships that could be construed as a potential conflict of interest.
